# Functional Feeding Groups of Aquatic Insects Influence Trace Element Accumulation: Findings for Filterers, Scrapers and Predators from the Po Basin

**DOI:** 10.3390/biology9090288

**Published:** 2020-09-14

**Authors:** Paolo Pastorino, Annalisa Zaccaroni, Alberto Doretto, Elisa Falasco, Marina Silvi, Alessandro Dondo, Antonia Concetta Elia, Marino Prearo, Francesca Bona

**Affiliations:** 1 The Veterinary Medical Research Institute for Piemonte, Liguria and Valle d’Aosta, Via Bologna 148, 10154 Torino, Italy; alessandro.dondo@izsto.it (A.D.); marino.prearo@izsto.it (M.P.); 2 Department of Veterinary Medical Science, University of Bologna, viale Vespucci 2, 47042 Cesenatico, Italy; annalisa.zaccaroni@unibo.it (A.Z.); marina.silvi@unibo.it (M.S.); 3 Department of Sciences and Technological Innovation, University of Piemonte Orientale, Viale Teresa Michel 11, Italy—ALPSTREAM Research Centre Ostana (CN), 15121 Alessandria, Italy; alberto.doretto@uniupo.it; 4 Department of Life Sciences, University of Torino, via Accademia Albertina 13, Italy—ALPSTREAM Research Centre Ostana (CN), 10123 Torino, Italy; elisa.falasco@unito.it (E.F.); francesca.bona@unito.it (F.B.); 5 Department of Chemistry Biology and Biotechnology, University of Perugia, via Elce di Sotto 8, 06123 Perugia, Italy; antonia.elia@unipg.it

**Keywords:** Heptageniidae, Hydropsychidae, metal accumulation, Odonata, water framework directive

## Abstract

For this study, we measured the concentrations of 23 trace elements (Al, As, Ba, Bi, Cd, Cr, Co, Cu, Fe, Ga, Hg, In, Li, Mn, Mo, Ni, Pb, Se, Sr, Ti, Tl, V, and Zn) in the whole bodies of three functional feeding groups (FFG) (filterers—Hydropsychidae, scrapers—Heptageniidae, and predators—Odonata) of aquatic insects collected from two sites in the Po basin (Po Settimo and Malone Front, Northwest Italy) to determine: (a) how FFG influence trace element accumulations, (b) if scrapers accumulate higher elements compared to the other FFG, since they graze on periphyton, which represents one of the major sinks of metals, and (c) the potential use of macroinvertebrates to assess the bioavailability of trace elements in freshwater. The hierarchical clustering analysis generated three main groups based on trace element concentrations: the most abundant elements were Fe and Al, followed by Sr, In, Zn, V, Mo, and Cu. Tl was below the limit of detection (LOD) in all FFG. Ga was detected only in scrapers from both sites and Hg only in predators from Po Settimo. The principal component analysis showed that concentrations of Al, As, Bi, Cd, Co, Cr, Ga, Fe, In, Mn, Pb, Ni, and Sr were highest in scrapers, suggesting that trace elements accumulate from the ingestion of epilithic periphyton (biofilm). Odonata (predators) accumulate certain elements (Ba, Hg, Li, Se, V, Ti, and Zn) in higher concentrations by food ingestion composed of different aquatic organisms. Differently, Cu and Mo concentrations were the highest in filterers due to their bioavailability in the water column. Non-metric multidimensional scaling clearly differentiated the FFG based on their ability to accumulate trace elements. The findings from this study represent an important step toward the definition of an innovative approach based on trace element accumulation by macroinvertebrates.

## 1. Introduction

Freshwater watercourses contain inorganic fractions of solutes in concentrations ranging from mg L^−1^ to µg L^−1^ or even less. These substances are derived from the dissolution of rocks or from solid or liquid atmospheric depositions [1]. Added to these natural vehicles of water enrichment is the anthropogenic input, which constitutes a major factor in environmental alteration [2,3]. Metal mining and industrial metal processing transfer huge masses of elements from the lithosphere (where they are immobilized in the mineral state) to the air, water, soil, and biosphere [4]. Alkaline elements (e.g., Na and K) and earthy alkaline (e.g., Ca and Mg) are present in larger quantities in freshwater. Their concentrations (order of mg L^−1^), together with those of the dissolved anions, pH, conductivity, hardness, and alkalinity, are the basic analytical parameters for the chemical characterization of water and are factors in the aqueous solubility equilibrium [5,6]. Additionally present in detectable concentrations and nearly constant over time are trace and rare earth elements. Their concentrations (generally an order of µg L^−1^) can be problematic for instrumental detection because of the wide variations in time and space [7]. Although their quantitative amounts may be relatively insignificant, their impacts on the environment and health are enormous [8,9].

Many trace elements, being constituents of organic molecules, are essential micronutrients for plants and animals, but their intakes in concentrations higher than required can result in intoxication inhibition phenomena (i.e., Cu, Fe, and Zn) [10,11]. Other elements are nonessential (i.e., Pb, Cd, and Hg), since they are not useful for organism function; however, they bind to molecules supplied as proteins and nucleic acids and denature them [12,13]. The concentration of trace elements in the environment has increased with the development of human activities such as mining, metalworking, fossil fuel consumption, and chemical compound production [14,15,16]. Awareness of the relationship between disease and exposure to these contaminants has grown with the discovery of new correlations between exposure and increased risk based on the greater frequency, duration, or extent of contact [17]. This has prompted greater attention to trace elements. National and international agencies have set up working groups, government commissions, and recommendations for the study and control of these contaminants (i.e., the foundation of the Trace Element Institute for the UNESCO Institute, active since 1996) [18].

As regards priority substances, the Water Framework Directive 2000/60/EC (WFD) of the European Parliament [19] imposes the use of biological elements for the classification of the ecological status of rivers; where data on the persistence and bioaccumulation are available, they should be considered to define the final value of the environmental quality standard. Global freshwater contamination due to anthropogenic activity or natural chemical compound uses is one of the key environmental issues facing humanity today [20,21]. The European Union has long recognized the importance of environmental monitoring for recording the exposure of the environment and humans to contaminants and the unique role monitoring instruments can play in identifying exposures to substances that pose risks for human health and the environment [22]. The monitoring of environmental contaminants using biota is based on the capacity of organisms to accumulate relatively large amounts of certain pollutants, even from highly diluted solutions, without obvious noxious effects [23]. The use of this type of monitoring is common in marine [24,25,26] and freshwater ecosystems [27]. Various freshwater organisms have been selected to assess trace elements in watercourses among which, fish are the most widely used [28] also for risk consumption [29]. Additionally, macroinvertebrates have begun to be used as a suitable matrix [7,30,31,32,33,34], since fish are absent from many polluted or unpolluted reaches of rivers, which limits the risk assessments of water and sediments [35].

Trace element concentrations in macroinvertebrates are directly related to the environmental levels [30,31,32,33,34,35,36]. Macroinvertebrates have a long vital cycle and are characterized by: differential adaptation to environmental alterations, limited mobility, and wide distribution [30,31,32,33,34,35,36,37]. Furthermore, the relatively ease of sampling and identification makes macroinvertebrates particularly suitable as tracers of trace elements in freshwater [35].

Macroinvertebrates can be grouped into functional feeding groups (FFG) according to the type of food resource that a taxon utilizes in an aquatic ecosystem [38,39]. The five major FFG are: scrapers (grazers), which consume benthic algae and associated materials, shredders, which consume leaf litter or other coarse particulate organic matter (CPOM), collectors-gatherers, which collect fine particulate organic matter (FPOM) from the stream bottom, filterers, which collect FPOM and dissolved organic matter (DOM) from the water column using a variety of filterers (DOM can also be acquired through an integumentary surface), and predators, which feed on other consumers [38]. The major routes of trace element uptakes in aquatic organisms are either directly from the water or indirectly through food [40,41,42].

Previous studies [31,32] found that collector-gatherers accumulate more metals than the other functional feeding groups, since their direct contact with sediments provide for greater uptakes of trace elements. However, those studies were focused only on a few heavy metals (e.g., As, Cd, Cr, Cu, Pb, and Zn) [31] and analyzed the correlation between the collector-gather density and trace element accumulation [32]. Therefore, few field data are available about other feeding strategies (filterers, scrapers, and predators). FFG are not always present or abundant simultaneously in all watercourses, since their relative abundance varies in response to natural gradients and anthropogenic pressures. For example, the presence of collector-gatherers and the absence of filterers or predators is correlated to highly polluted areas [43,44]. The aim of the present study was to determine trace element accumulations in aquatic insects of three FFG (predators, filterers, and scrapers) from two sites in the Po basin (Northwest Italy) to determine: (a) how FFG influence trace element accumulations, (b) if scrapers accumulate higher elements compared to the other FFG, since they graze on periphyton, which represents one of the major reservoirs and sinks for many metals [45], and (c) the potential use of macroinvertebrates to assess the bioavailability of trace elements in freshwater.

## 2. Materials and Methods

### 2.1. Study Area

For the purpose of this study, two sites in the Po basin (Northwest Italy) were selected and sampled in March 2018, since both are inhabited by the same FFG (predators, filterers, and scrapers) of the same order or family.

Site 1 (Po Settimo) is located on the Po river, downstream the Turin Metropolitan Area; the valley is characterized by extensive farming and intense mining and by the discharge of civil and industrial wastewater. Physicochemical parameters were (March 2018) mean values: T 11.3 °C, pH 7.5, conductivity 287 µS cm^−1^, and dissolved oxygen 7.8 mg L^−1^). The ecological status (sensu WFD) was classified as “moderate” [46]. The riverbed is largely disconnected from the perifluvial belt, as it is intensely anthropized and urbanized. Other important manmade elements are the bank defenses that stabilize the riverbed and the numerous bridges. The site receives water also from two tributaries: the Dora Riparia and the Stura di Lanzo. These pressures result in a high concentration of effluent from industrial, zootechnical, and agricultural activities that negatively affect the water quality. Site 2 (Malone Front) is located in the Municipality of Front (Turin Province), on the river Malone, which is a Po tributary. The main sources of pollution are civil wastewater discharge and effluents of agricultural and industrial origins. Physicochemical parameters were (March 2018): mean values: T 11.1 °C, pH 7.2, conductivity 253 µS cm^−1^, and dissolved oxygen 8.7 mg L^−1^. The ecological status of this site was “moderate” [46].

### 2.2. Sampling of Macrobenthic Invertebrates

Macrobenthic invertebrates were sampled using a Surber net set up in a wadable stretch at each site [47,48] and selected based on their abundance to have enough biomass for a trace element analysis. All collected organisms were sorted in the field, counted, and identified to the order or family level. For each taxon, 100–120 individuals were collected per site, and individuals were pooled to obtain three replicates (30–40 individuals). The attempt was made to sample larger individuals to minimize differences in metal concentrations due to size [35]. This procedure also minimized differences in diet compositions, which could vary with age [49]. Functional feeding groups (FFG) were assigned to each taxon, as described in Merritt and Cummins [50]. Samples were transported refrigerated to the laboratory and immediately stored at −20 °C until trace element analysis.

### 2.3. Trace Element Analysis

In each site, 23 trace elements (Al, As, Ba, Bi, Cd, Cr, Co, Cu, Fe, Ga, Hg, In, Li, Mn, Mo, Ni, Pb, Se, Sr, Ti, Tl, V, and Zn) were detected in the whole bodies of macrobenthic invertebrates [35] by inductively coupled plasma-optic emission spectrometry (ICP-OES) using a Perkin Elmer Optima 2100 DV instrument (PerkinElmer, Inc., Shelton, CT, USA), coupled with a CETAC U5000AT+ ultrasound nebulizer (Cetac Technologies, Inc., Omaha, NE, USA) for mercury. All these elements can affect organisms; thus, it is important to assess their bioavailability in freshwater [30]. We analyzed the whole-body concentrations (gut contents included), since analysis of tissue concentrations alone does not allow for the detection of trace elements in sites with very low metal concentrations [51].

All samples were homogenized and microwave-digested using a Milestone ETHOS ONE oven using 4-mL nitric acid and 1-mL hydrogen peroxide. All reagents were from Merck, Darmstadt (Germany); acids were of Suprapur grade [52]. Analytical results are reported as ug g^−1^ wet weight (w.w.). Quality assurance tests performed during analysis included the recovery rate and blank and certified material analyses; all quality results were within acceptable ranges. Table S1 presents the limit of detection (LOD), the reference material values, and the percentages of recovery.

### 2.4. Statistical Analysis

The Kolmogorov-Smirnov test was used to test the normality. The validity of the homogeneous variance’s assumption was investigated by a Bartlett’s test. The differences in trace element concentrations (predator, scrapers, and filterers) from each site were tested using the Kruskal-Wallis test (followed by the Conover-Iman post-hoc test) or the Mann-Whitney U test if the elements in one of the sampled FFG showed a concentration < LOD. Trends in trace element concentrations between the FFG from the two sampling sites were evaluated by principal component analysis (PCA). Agglomerative hierarchical clustering analysis (AHC) using Euclidean distance as a measure of similarity was applied to organize trace elements into groups based on their total concentrations (considering the three FFG) at the two sampling sites. Non-metric multidimensional scaling (NMDS) with a Bray-Curtis dissimilarity matrix was performed to summarize the dissimilarity in trace element accumulations in the FFG. The criterion for significance was set at *p* < 0.05. Statistical analyses were performed using RStudio^®^ version 1.1.463 (RStudio, Inc., Boston, MA, USA).

## 3. Results

**Figure 1:**
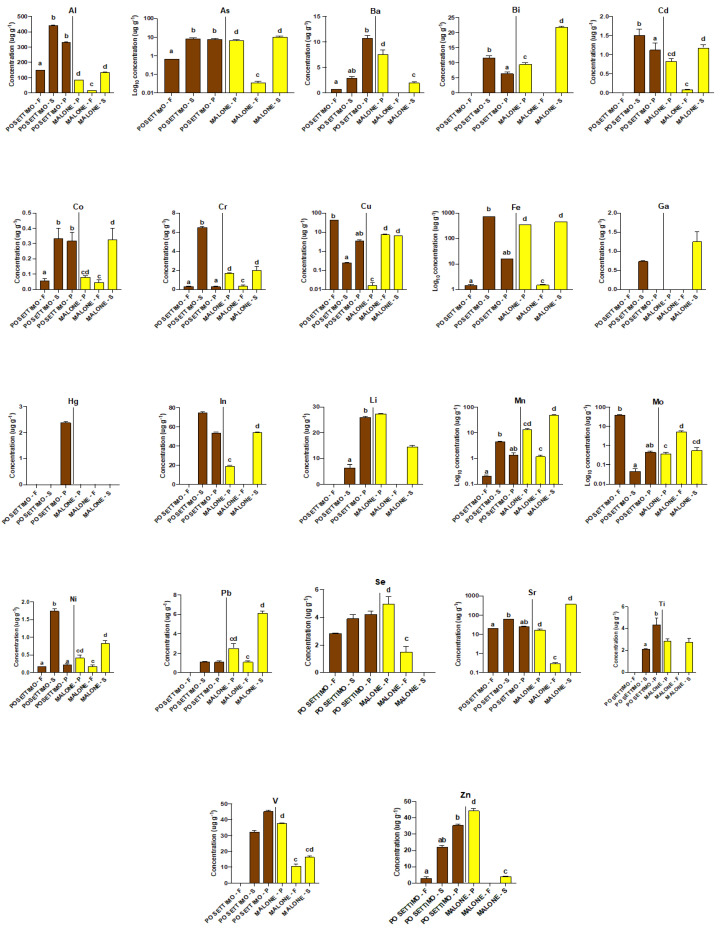
Bar graphs (mean ± standard deviation) of trace element concentrations (µg g^−1^) of filterers (F), scrapers (S), and predators (P) from the Po Settimo (brown) and the Malone (yellow) site. Lowercase letters denote differences revealed by Conover-Iman post-hoc or Mann-Whitney tests among the three functional feeding groups at each site: Po Settimo (a,b) and Malone Front (c,d).

At both sites, we captured individuals belonging to the family Heptageniidae (Ephemeroptera; FFG scrapers) and Hydropsychidae (Trichoptera; FFG filterers) and order Odonata (FFG predators). Figure 1 shows the bar graphs (mean ± standard deviation) for each element detected in the three FFG from the two sites. Tl was below the limit of detection (LOD) in all FFG. Ga was detected only in the scrapers from both sites, while Hg only in the predators from Po Settimo (Table S2). The Kruskal-Wallis test revealed a significant difference (*p* < 0.05) in trace element concentrations (Al, As, Co, Cr, Cu, Fe, Mn, Mo, Ni, and Sr) between the FFG from both sites; in two elements (Ba and Zn) from Po Settimo; and in three elements (Cd, Pb, and V) from Malone. The Conover-Iman post-hoc test showed significantly higher concentrations (*p* < 0.05) in both scrapers and predators compared to filterers for two elements (Al and Co) from Po Settimo, for two elements (Fe and Sr) from Malone, and for two elements (As and Cr) from both sites. Significantly higher concentrations were also recorded (Conover-Iman test; *p* < 0.05): (a) in predators compared to filterers (Ba and Zn) from Po Settimo and (V) from Malone; (b) in scrapers compared to filterers (Fe and Sr) from Po Settimo, (Cd, Co, Ni, and Pb) from Malone, and (Mn) from both sites; (c) in scrapers compared to both predators and filterers (Cr and Ni) from Po Settimo; (d) in filterers compared to scrapers (Mo) from Po Settimo; and (e) in filterers compared to predators (Mo) from Malone. The Mann-Whitney U test revealed significant differences in trace element concentrations between predators and scrapers (Bi) from both sites (*p* < 0.05); (Ba, In, and Zn) from Malone (*p* < 0.05); (Cd, Li, and Ti) from Po Settimo (*p* < 0.05); and between predators and filterers (Se) from Malone.

**Figure 2:**
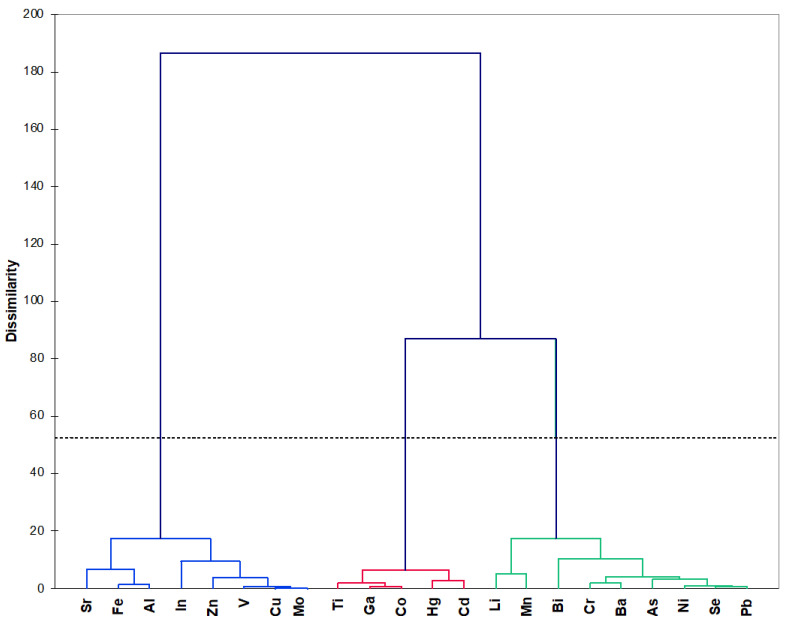
Dendrogram generated by hierarchical clustering analysis. The dotted line represents automatic truncation, resulting in three groups: group 1 (blue), group 2 (red), and group 3 (green).

AHC produced three clusters generated based on trace element concentrations at the two sampling sites (Figure 2). Within-group similarity was maximized and among-group similarity was minimized, indicating a relatively high independency for each cluster: cluster 1 (blue) grouped Sr, Fr, Al, In, Zn, V, Cu, and Mo; cluster 2 (red) grouped Ti, Ga, Co, Hg, and Cd; and cluster 3 (green) grouped Li, Mn, Bi, Cr, Ba, As, Ni, Se, and Pb. Two groups (1 and 3) were approximately the same size, and the second had only five elements. The second group (red) was more homogeneous than the third group (flatter on the dendrogram), since it contained trace elements present at lower concentrations compared to the other two groups.

**Figure 3:**
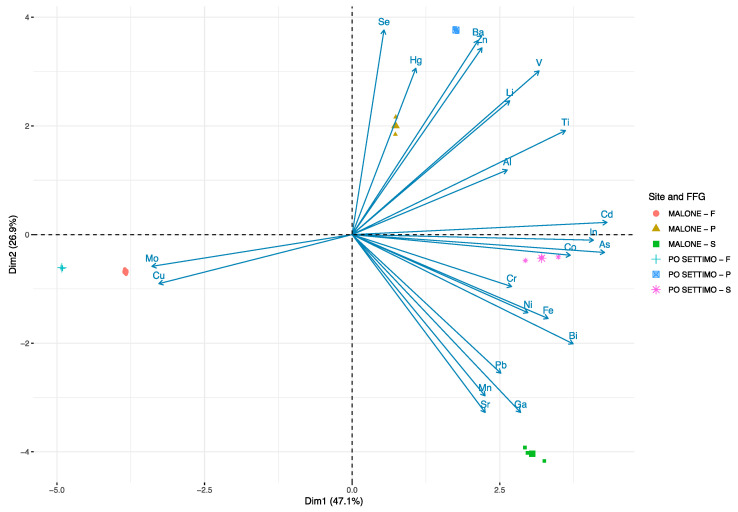
Biplot of scores and loadings from the principal component analysis (PCA). The scores of each functional feeding group (F = filterers, P = predators, and S = scrapers) from each site (Po Settimo and Malone) are denoted by color and a symbol (largest symbol = average value).

The PCA results (Figure 3) showed that the first (PC1) and the second (PC2) components accounted for meaningful amounts of the total variance (74%): PC1 explained 47.1% of the total variance and was positively correlated with As, Bi, Cd, Co, Cr, Fe, In, Li, Ni, Ti, and V and negatively correlated with Cu and Mo. PC2 explained 26.9% of the total variance and was positively correlated with Ba, Hg, Se, and Zn and negatively correlated with Ga, Pb, Mn, and Sr.

The FFG from each site are arranged according to trace element concentrations measured at the two sites. There is a clear separation between each FFG: predators are located in the upper-right quadrant of the plot in relation to higher concentrations of Ba, Hg, Li, Se, V, Ti, and Zn; scrapers are located in the lower-right quadrant of the plot in relation to higher concentrations of Al, As, Bi, Cd, Co, Cr, Ga, Fe, In, Mn, Pb, Ni, and Sr, while filterers are located on the left side in relation to higher concentrations of Cu and Mo.

**Figure 4:**
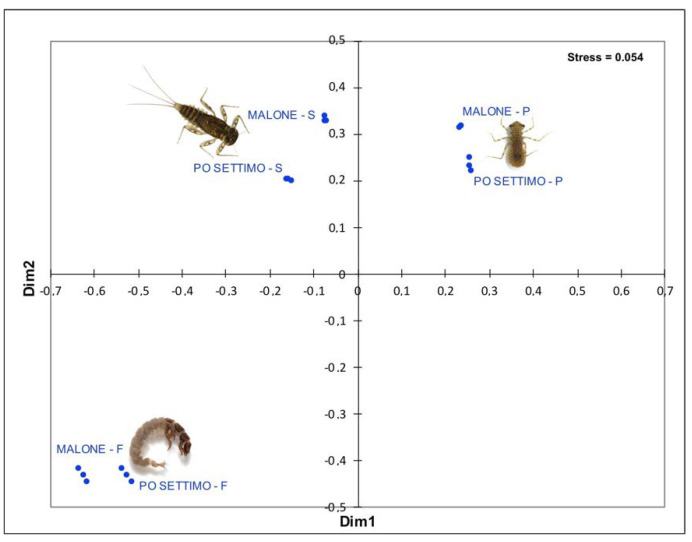
Non-metric multidimensional scaling (NMDS) on functional feeding groups (FFG) from the Po Settimo and Malone sampling sites. The upper-case letters denote FFG: F = filterers, P = predators, and S = scrapers.

NMDS clearly showed a separation of the FFG based on their ability to accumulate trace elements (Figure 4). Each FFG is well-separated from the other. The stress value was 0.052; thus, the NMDS plot was considered to be a good representation [53].

## 4. Discussion

Metal contaminations in macroinvertebrates usually result in fewer sensitive taxa and less species diversity. The Heptageniidae family is particularly sensitive to heavy metals [54], and its occurrence in both the Po Settimo and the Malone Front sites suggested that the sites have low metal contaminations. For this study, we recorded also the Hydropsychidae family, which is considered a good bioindicator of environmental pollution, since it is widely distributed, abundant, resistant to high variations in water quality, tolerant to metal pollution, and many other disturbances [55,56,57]. Furthermore, we considered Odonata, since it gives a rapid and sensitive response to accumulations of trace elements [9]. The large size of our selected taxa (also in terms of biomass) make them perfect for chemical analysis and useful for evaluating metal bioaccumulations [51].

Comparisons of the trace elements in the FFG from both sites showed that iron and aluminum were the two elements with the highest concentrations, followed by strontium, indium, zinc, and vanadium. Iron is the most common element in the Earth’s crust and can be found in both the ferrous (Fe^2+^) and the ferric (Fe^3+^) states or in other forms from wastewaters [58]. Fe is an essential element for the physiology of freshwater organisms [59]. Our data are in-line with those reported by Pastorino et al. [32] for macrobenthic communities from six watercourses in Northeast Italy. In our study, the Fe concentration was highest in the scrapers and lowest in the filterers from both sites. Our data for filterers are lower than those reported by Maramis and Kristijanto [60] (range, 4.65–15.83 µg g^−1^) recorded for the genus *Hydropsyche* from the Kreo River (Hungary).

The element with the second-highest concentration in our samples was aluminum. Although the most abundant metallic element in the lithosphere [61], it has little or no biological functions for organisms, and its toxicity is greatest in acid water, with a maximum toxicity of around pH 5.0 to 5.2 [62]. Additionally, the Al concentration was higher in the scrapers than the other two FFG from both sampling sites. Compared to our data, a previous study reported higher Al concentrations (range, 1240–2110 µg g^−1^) for *Hydropsyche* [55], which were recorded for the Sacramento River (California, USA), a watercourse affected by acid mine drainage.

Strontium occurs in different freshwater compartments, and several of its compounds are dissolved in water [63]. The high concentrations recorded at our sampling sites is probably related to human activities (i.e., TV screen manufacture), since its usage is similar to that of calcium and barium [64]. The Sr concentration was higher in scrapers compared to the other two FFG from both sampling sites. To our best knowledge, there are no previously published works on Sr accumulation by macrobenthos with which to compare our data.

Generally, indium is not widely present in aquatic environments [65]; nonetheless, we found notable concentrations in our samples, suggesting that it is becoming an emerging contaminant. It is employed in the manufacture of integrated circuits and photoelectric equipment [65]. Indium concentrations were higher in scrapers compared to predators from both Malone and Po Settimo but undetectable in filterers, probably due to the insoluble nature of its compounds (i.e., oxide) [66,67].

Zinc occurs naturally in the environment; high concentrations in aquatic environments are due to industrial activities, mining, coal and waste combustions, and steel processing [68]. Rainbow et al. [57] studied the Zn concentration in 24 watercourses in Southwest England and found levels in *Hydropsyche* spp. ranging from 203 to 600 µg g^−1^, much higher than our data. In our samples, the predators accumulated more Zn than the scrapers or the filterers. These observations were corroborated by Santoro et al. [31] for macrobenthic invertebrates from the Basento River (Italy).

Vanadium was higher in predators compared to scrapers from both sampling sites; contamination is usually caused by fossil fuel and coal combustion, the use of fertilizers, and pesticides [69]. Vanadium appears to be necessary for human health, but a specific function in aquatic invertebrates has not yet been found [70].

Regarding the other trace elements, we found that Heptageniidae and Odonata accumulated more trace elements compared to Hydropsychidae for all elements, except for Cu and Mo, which were significantly higher in Hydropsychidae from both sites. This difference indicates the potential solubilization of their compounds in water based on their chemical structures, water pH, temperatures, and alkalinity. Readily soluble in water, molybdate compounds such as ammonium and sodium molybdate are used in ceramic glazes and pigments [71]. Additionally, Cu dissolved in water, as the Cu^2+^ ion is the most available and toxic form in freshwater [72].

Whole-body concentrations in macroinvertebrates, although higher than tissue concentrations (without gut contents), can be used to indicate environmental pollution [51], since they provide a measure of bioavailable metal concentrations in the environment [73]. The internal distribution of metals in body tissues is very heterogeneous, and the distribution patterns tend to be both metal and taxon-specific [74]. Aquatic insects can bind trace elements on the surface of their chitinous exoskeleton and/or incorporate them into body tissues [75]; thus, detecting their concentrations in the whole body is the best solution. On this path, Fränzle [76] found that insect chitin can intercept both toxic (i.e., Pb, Sb, and Cd) and essential trace elements (i.e., Cu, Fe, and Zn) from different environmental compartments.

Metal bioavailability is influenced by several factors (i.e., pH, temperature, redox potential, and total organic content) and is the proportion of total metals that are available for incorporation into the biota [9,77]. For benthic organisms, the efficiency of bioaccumulation is related also to the geochemical characteristics of the sediments [77]: metals bound to iron and manganese oxyhydroxides or sulfides (i.e., As) are less available than those forming carbonate salts or are bound by ionic exchange (i.e., Cd and Zn) [9,78]. Collector-gatherer feeders (i.e., Oligochaeta, Diptera, and Chironomidae) are excellent accumulators [34], since they move into sediment and collect smaller particles of organic matter, entering in contact with metal-polluted substrates [31]. Furthermore, they are abundant in fine and coarse sediments in rivers and streams [31,79]. Thus, they are suitable for bioaccumulation studies [7,30,32,79]. Metal accumulations in *Chironomus* sp. and Tubificidae (Oligochaeta) have the potential to be used also as predictors of ecological effects in aquatic ecosystems [80].

Macroinvertebrates are exposed to metals through their gills and by dietary pathways through water filtration (filterers), grazing on periphyton (scrapers), or preying on other invertebrates (predators) [81]. In our study, although the scrapers accumulated higher amounts of trace elements (Al, As, Bi, Co, Cd, Cr, Ga, Fe, In, Mn, Pb, Ni, and Sr) compared to the other FFG, the concentrations of certain elements were higher for the predators (Ba, Hg, Li, Se, V, Ti, and Zn) and for the filterers (Mo and Cu) compared to the scrapers.

The amounts of trace elements in macroinvertebrates are closely related to the metal uptake, transport, utilization, and excretion, which vary by species [82]. The amount of trace metals accumulated by an individual reflects the net balance between the rate of metal influx from both dissolved and particulate sources and the rate of metal efflux from the organism [76]. Furthermore, the concentrations of trace elements in members of a species living at the same time and place can differ depending on the metal in question and on the size, age, sex, and developmental stage of the individuals. Aquatic insects can accumulate metals directly from the sediments or by food ingestion [82]; for such reasons, it is important to consider the feeding strategies of insects [31].

Aquatic invertebrates within closely related taxa, down to species in the same genus living in the same habitat, may well have very different body concentrations of trace metals [59,75]. For example, Fletcher et al. [83] studied the trace element accumulations in eight genera of lotic dragonfly nymphs, revealing both a generic and intrageneric variation in accumulation patterns. Furthermore, the same authors found that the accumulations of some trace elements differed significantly among dragonflies that were different in body forms. Otherwise, *Hydropsyche* species (*H. cockerelli* and *H. occidentalis*) showed similar bioaccumulation patterns in several sites from Montana, USA [84,85]. However, Awrahman et al. [86] highlighted how body mass influenced the accumulations of As, Cu, Pb, and Zn in *Hydropsyche siltalai* and *H. angustipennis*. As regarding Ephemeroptera, Fialkowski et al. [87] showed how *Baetis rhodani* and *B. verus* collected in river sites from Poland did not show significant differences in metal accumulations. The consideration of such studies may be necessary when using macroinvertebrates to assess the bioavailability of trace elements, avoiding errors in the analysis and conclusions in biomonitoring programs.

Our findings seem to suggest that feeding on periphyton by Heptageniidae is the best route to assess the bioavailability of certain elements but not all. Indeed, Odonata biomagnify Ba, Hg, Li, Se, V, Ti, and Zn, since they are located higher in the trophic chain. On the other hand, Hydropsychidae are good indicators of Cu and Mo, due to their bioavailability in the water column.

Odonata can accumulate metals in high concentrations by food ingestion composed of different aquatic organisms [88]. Previous field studies [89,90,91] showed that epilithic periphyton (biofilm) is the major sink of metals and that it accumulates more metals than sediments in certain cases [45,92], which explains the higher concentrations we recorded for the scrapers. Furthermore, the siltation (fine sediment deposition) on periphyton [93] could also promote metal accumulations by scrapers, since they could also ingest deposited FPOM, increasing their whole-body metal contents.

## 5. Conclusions

The European Water Framework Directive [19] has recognized biota as suitable matrices for monitoring long-term changes in water quality [94,95]. The national water authorities should provide field data based on biota for the analysis of priority substances (heavy metals included), as required by Directive 2013/39/EU [96]. With this study, we provide new field data from two lowland sites that illustrate the potential use of macroinvertebrates to assess the bioavailability of trace elements in freshwater. Our study suggested that individuals belonging to Heptageniidae are not completely adequate to assess the bioavailability of some elements, such as Zn, Cu, and Hg, which are very important metals largely recognized to be harmful to organisms. If the Heptageniidae, Hydropsychidae and Odonata are not present simultaneously, other families or FFG (i.e., chironomids or oligochaetes) should be used. Thus, it can be concluded that organisms from different FFG should be used in parallel to cover the bioavailability of all metals.

A comparison study on the bioaccumulation of trace elements between scrapers and collector-gatherers is needed in order to determine the best bioindicators for assessing the bioavailability of metals in freshwater and identify the limitations for using each of these two FFG.

Furthermore, future research is necessary to identify the best family within scrapers to assess the bioavailability of all metals. Complementary studies should also be conducted to determine the best family/group within filterers and predators for bioaccumulation studies.

The results obtained in this present paper represent an important step toward the definition of an innovative approach based on trace element accumulations by macroinvertebrates.

## Supplementary Materials

The following are available online at https://www.mdpi.com/2079-7737/9/9/288/s1: Table S1: Limit of detection (LOD) (µg g^−1^), reference material values, and percentages of recovery. Table S2: Concentrations of trace elements (ug g^−1^ wet weight) in macroinvertebrates (S = scrapers, F = filterers, and P = predators) from Po Settimo and Malone. LOD = limit of detection and SD = standard deviation.
