# Supplementary material for: Functional Feeding Groups of Aquatic Insects Influence Trace Element Accumulation: Findings for Filterers, Scrapers and Predators from the Po Basin
Source: Biology (Basel). 2020 Sep 14;9(9):288. doi: 10.3390/biology9090288 (PMC7564872; doi:10.3390/biology9090288)
Supplement: Supplementary file 1 [file biology-09-00288-s001.pdf]

Table S1. Limit of Detection (LOD) (μg g^{-1}), reference material values and percentages of recovery.

Element	LOD	Reference Material	% Recovery
Al	0.014	HISS-1 Marine Sediment Reference Materials for Trace Metals and other Constituents	101
As	0.021	HISS-1 Marine Sediment Reference Materials for Trace Metals and other Constituents	98
Ba	0.063	-	/
Bi	0.007	-	/
Cd	0.0015	HISS-1 Marine Sediment Reference Materials for Trace Metals and other Constituents	103
Co	0.0014	HISS-1 Marine Sediment Reference Materials for Trace Metals and other Constituents	93
Cr	0.0012	HISS-1 Marine Sediment Reference Materials for Trace Metals and other Constituents	97
Cu	0.0003	HISS-1 Marine Sediment Reference Materials for Trace Metals and other Constituents	99
Fe	0.00072	HISS-1 Marine Sediment Reference Materials for Trace Metals and other Constituents	101
Ga	0.0014	-	/
Hg	0.0004	HISS-1 Marine Sediment Reference Materials for Trace Metals and other Constituents	97
In	0.001	-	/
Li	0.00032	HISS-1 Marine Sediment Reference Materials for Trace Metals and other Constituents	96
Mn	0.00032	HISS-1 Marine Sediment Reference Materials for Trace Metals and other Constituents	104
Mo	0.0005	HISS-1 Marine Sediment Reference Materials for	95

Element	LOD	Reference Material	% Recovery
		Trace Metals and other Constituents	
Ni	0.0028	HISS-1 Marine Sediment Reference Materials for Trace Metals and other Constituents	110
Pb	0.013	HISS-1 Marine Sediment Reference Materials for Trace Metals and other Constituents	94
Se	0.00014	HISS-1 Marine Sediment Reference Materials for Trace Metals and other Constituents	103
Sr	0.0005	HISS-1 Marine Sediment Reference Materials for Trace Metals and other Constituents	97
Ti	0.0002	HISS-1 Marine Sediment Reference Materials for Trace Metals and other Constituents	92
Tl	0.002	HISS-1 Marine Sediment Reference Materials for Trace Metals and other Constituents	93
V	0.00084	HISS-1 Marine Sediment Reference Materials for Trace Metals and other Constituents	98
Zn	0.00072	HISS-1 Marine Sediment Reference Materials for Trace Metals and other Constituents	113

Table S2. Concentration of trace elements (ug g^{-1} wet weight) in macroinvertebrates (S = scrapers; F = filterers; P = predators) from Po Settimo and Malone. LOD = limit of detection; SD = standard deviation.

	PO SETTIMO - S		PO SETTIMO - F		PO SETTIMO - P		MALONE - F		MALONE - P		MALONE - S	
	**Mean**	**SD**	**Mean**	**SD**	**Mean**	**SD**	**Mean**	**SD**	**Mean**	**SD**	**Mean**	**SD**
**Al**	444.32	1.12	149.42	1.54	332.44	1.15	17.13	0.15	85.52	0.65	136.16	0.96
**As**	8.32	0.87	0.66	0.01	7.73	0.78	0.04	0.01	6.60	0.94	9.52	0.99
**Ba**	2.93	0.24	0.78	0.02	10.71	0.62	<LOD	-	7.52	0.95	2.01	0.16
**Bi**	11.58	0.69	<LOD	-	6.40	0.45	<LOD	-	9.52	0.50	21.72	0.42
**Cd**	1.51	0.15	<LOD	-	1.13	0.18	0.08	0.01	0.83	0.08	1.18	0.09
**Co**	0.33	0.07	0.06	0.02	0.32	0.06	0.05	0.02	0.08	0.01	0.33	0.08
**Cr**	6.49	0.10	0.31	0.02	0.29	0.09	0.37	0.09	1.70	0.05	2.02	0.38
**Cu**	0.25	0.01	42.04	0.83	3.55	0.42	7.51	0.38	0.02	0.01	6.41	0.19
**Fe**	722.87	6.25	1.47	0.09	16.09	0.16	1.52	0.06	349.18	1.00	456.73	1.35
**Ga**	0.73	0.01	<LOD	-	0.00	0.00	<LOD	-	<LOD	-	1.24	0.26
**Hg**	<LOD	-	<LOD	-	2.38	0.04	<LOD	-	<LOD	-	<LOD	-
**In**	74.82	1.16	<LOD	-	53.75	0.90	<LOD	-	19.37	0.73	54.07	0.70
**Li**	6.50	1.10	<LOD	-	26.13	0.26	<LOD	-	27.31	0.08	14.57	0.58
**Mn**	4.58	0.12	0.21	0.02	1.38	0.29	1.21	0.12	13.46	1.14	50.80	1.00
**Mo**	0.05	0.02	39.20	1.02	0.44	0.06	5.39	0.70	0.37	0.09	0.55	0.26
**Ni**	1.75	0.06	0.16	0.01	0.23	0.02	0.18	0.04	0.42	0.08	0.83	0.07
**Pb**	1.14	0.05	<LOD	-	1.10	0.10	1.10	0.10	2.50	0.50	6.12	0.23
**Se**	3.91	0.31	2.83	0.07	4.20	0.24	1.49	0.42	4.98	0.53	<LOD	-
**Sr**	61.15	0.90	20.16	0.10	25.06	1.07	0.29	0.05	17.11	0.99	374.80	1.65
**Ti**	2.16	0.01	<LOD	-	4.34	0.60	<LOD		2.88	0.20	2.74	0.35
**V**	32.91	1.03	<LOD	-	45.27	0.85	10.79	1.10	37.91	0.20	16.45	0.50
**Zn**	22.07	1.00	2.89	0.94	35.51	0.81	<LOD	-	44.36	1.50	4.15	0.15
